# The field efficacy of *Nigella sativa* and *Berberis vulgaris* methanolic extracts against *Haemoproteus columbae*

**Published:** 2018

**Authors:** Seyed Mostafa Razavi, Mohammad Asadpour, Seyed Hossein Malekpour, Arash Jafari

**Affiliations:** *Department of Pathobiology, School of Veterinary Medicine, Shiraz University, Shiraz, Iran*

**Keywords:** Nigella sativa, Berberis vulgaris, Haemoproteus columbae, Pigeon

## Abstract

**Objective:**

The methanolic extracts of *Nigella sativa*L. seeds (MENS) and *Berberis vulgaris *L.(MEBV) were investigated for treatment of *Haemoproteus columbae-*infected pigeons (*Columba livia domestica*).

**Materials and Methods:**

One hundred twenty naturally-infected pigeons were randomly divided into four groups of thirty each. Two groups were treated separately with the extracts, while the positive and negative control groups were given buparvaquone (*Butalex*®) and distilled water, respectively. The parasitaemia rate was calculated in all groups before and after the experiment at four-day intervals for16 days.

**Results:**

The results showed a high therapeutic effect for MENS with a progressive decrease in average parasitaemia rate from 18.17% before treatment to 0.73% at the end of treatment (p<0.05), while *Butalex*® was able to suppress the parasitemia rate from 18.90% before treatment to 0.23% at the end of experiment (p<0.05). However, no significant changes in parasitemia rate were evident in groups treated with MEBV (p>0.05).

**Conclusion:**

Methanolic extracts of *N. sativa *showed therapeutic effects against *H. columbae *and may be regarded as a suitable choice for further studies to develop new drugs against blood parasites, in both animals and human beings.

## Introduction

Infectious diseases threaten the health and survival of domesticated and wildlife populations around the world. Haemosporida of the genera *Haemoproteus* and *Plasmodium* (Phylum Apicomplexa, Order Haemosporida, class Sporozoa) are relatively well known. These parasites are common vector-borne blood parasites with a worldwidedistribution, which are transmitted to a wide variety of avian species. *Plasmodium* is genetically closely related to *Haemoproteus*, but there are differences in their life cycles and primary vectors (Martinsen et al., 2008 ▶). *H.columbae, *which is also known as pigeon malaria, is transmitted to pigeons by pigeon louse fly,*Pseudolynchiacanariensis (*Order*Diptera*, Family*Hippoboscidae)*, which transmit the disease by inoculating the infective sporozoites. Schizogony occurs in lung endothelium and the merozoites are released; then, merozoites invade erythrocytes and develop into gametocytes. Gametocytes are visible in blood smears and partially surround the nucleus of RBC (Bishopp, 1929 ▶; Valkiunas, 2004 ▶). Some researchers have considered *Haemoproteus spp.* infection a mild or even nonpathogenic parasite in birds (Ashford, 1971 ▶; Garvin et al., 2003 ▶). Nowadays, it is well understood that *Haemoproteus* can affect avian body condition (Valkiūnas et al., 2006 ▶), immune and reproductive systems (Tomás et al., 2007 ▶), and community relationships (Ricklefs et al., 2004 ▶) and may lead to death or extinction of more susceptible bird species (Atkinson et al., 2000 ▶). An experiment done by Garvin et al. (2003) ▶ on pathologic effects of *Haemoproteus*-induced infection indicated that the erythrocytic form causes severe anemia (Cardona et al., 2002 ▶; O'roke, 1930 ▶), weakness and anorexia (Garvin et al., 2003 ▶). Also, Manwell and Loeffler (1961) ▶ revealed that the erythrocytic phase of *H.columbae* can consume glucose even 100 times more than that of uninfected RBC. Another investigation demonstrated that blood parasites such as *Haemoproteus* are common causes of death and reduce avian survival by increasing the predation risk under natural conditions (Møller and Nielsen, 2007 ▶). *Plasmodium* and *Haemoproteus* are commonly used as models for hypotheses evaluation in ecology (Knowles et al., 2010b ▶; Ricklefs et al., 2005 ▶) and also for investigation of diagnosis and control strategies for human malaria (Slater, 2005 ▶). Resistance of some blood parasites to standard drugs has motivated scientists to introduce more effective drugs with novel modes of action (Muregi et al., 2003 ▶). Therefore, studies on appropriate alternative compounds for development of new treatment strategies are needed. Herbal extracts (e.g. quinine and artemisinin) have been a valuable source of new drugs, especially anti-haemosporidial agents (Gessler et al., 1994 ▶). Considerable evidences have demonstrated that some plant products can be useful as anti-haemosporidial agents (Muregi et al., 2003 ▶; Okeola et al., 2011 ▶; Rodrigues and Gamboa, 2009 ▶). To the best of our knowledge, no documented research has studied the effects of plant extracts against*H.columbae*. *Nigella sativa* L.*, *commonly known as black seed, belongs to the *Ranunculaceae* family. This seed has been used in Middle and Far East communities as a natural drug for treatment of many diseases. Previously, *N. sativa *(*“SiahDaneh” *in Persian) was used as a drug for treatment of tumor (Ahmad et al., 2013 ▶), diabetes (El-Shabrawy and Nada, 1996), and cestode and nematode infections (Mahmoud et al., 2002 ▶). Furthermore, its methanolic extracts have shown antimalarial, antioxidant and anti-leishmanial activities, which were more effective than chloroquine in parasite clearance (Mahmoudvand et al., 2014a ▶; Okeola et al., 2011b ▶). Moreover, *Berberisvulgaris L. *called “*zereshk*” (a Persian name for the dried fruit of *Berberis*) is another desirable plant which showed high anti-leishmanial activity in BALB/c mice and culture models (Mahmoudvand et al., 2014 ▶; Salehabadi et al., 2014 ▶). Today, application of plants such as *N. sativa* and *B. vulgaris *are becoming very popular, some of them have been employed for decades to treat different infectious agents (Okeola et al., 2011 ▶; Salehabadi et al., 2014 ▶)*. *The objective of the present study was to examine the therapeutic potential of *N. sativa *and *B. vulgaris *methanolic extracts in domesticated pigeons (*Columba liviadomestica*) naturally infected with *H.columbae*.

## Materials and Methods

**Plant materials**

*N. sativa* seeds and dried *B. vulgaris *fruit were purchased from a local herbal market in Shiraz (Iran). The taxonomic identity of each plant was authenticated (*N. sativa*: NREF-96-201 and *B. vulgaris*: NREF-96-202) by F. Bahmanzadegan and M. Etemadi (Research Center of Agriculture, Natural Resources and Education, Fars Province, Iran). Methanolic extract of two plants were obtained according to the method previously described by Moazeni and Nazer (2010). Briefly, plants were dried under shade, and powdered mechanically using a commercial electrical blender. To obtain the methanolic extract of each plant, 500 g of powder was added to 1 liter of methanol and mixed gently for 1 hr using a magnetic stirrer. The obtained mixture was left at room temperature for 24 hr. The mixture was stirred again and ﬁltered and then the solvent was removed by evaporation in a rotary evaporator. The obtained residue was placed into a sterile glass container and stored in the refrigerator at 4ºC for later use. Approximately 11 g of dried extracts from each 500 gr of dried powder of both plants were obtained.

**Animals**

The present study was carried out on a flock of domesticated pigeons (*Columba liviadomestica*) in Shiraz, Fars province, Iran. Blood smears were obtained from all the flock. One hundred twenty female pigeons, between nine months and one year old, weighing 450–500 g, and naturally infected with *H. columbae*were chosen for the present experiment and randomly divided into different groups. The pigeons were not treated before experiment.

**Parasitemia rate**

Blood samples were obtained from the brachial vein punctured by a lancet and smears were prepared on clean microscopic slides, fixed by absolute methanol, and then stained with 10% aqueous Giemsa stain for 45 min. The number of parasitized red blood cells containing halter-shaped gametocyte in each smear was counted from at least 600 RBC and the parasitemia rate in each sample, was monitored at four-day intervals for 16 days (Ishtiaq et al., 2007 ▶; Salehabadi et al., 2014 ▶).

**Field application and study design**

The infected pigeons were randomly divided into four groups of 30animals each. The experimental groups were separately kept in cages with free access to food and water. Three naturally infected groups were treated with *N.sativa*seeds, dried *B. vulgaris *methanolic extract and *Butalex*^®^ (positive control group) and the negative control group was treated with distilled water. Methanolic extracts of *N. sativa* seeds (MENS) was administered at a dose of 12.5% based on the previous studies (Al-Naggar et al., 2003 ▶; Okeola et al., 2011 ▶) and our preliminary study (data not shown). The recommended dose for *B.vulgaris*methanolic extract (MEBV) was 20% (Salehabadi et al., 2014 ▶). Both extracts were administered once a day by oral gavage for 16 days. *Butalex*^®^ was administered intramuscularly (IM) as a single recommended dose (El-Metenawy, 1999 ▶). Parasitemia rate was calculated for all groups on days 4, 8, 12, 16.

**Statistical analysis**

Statistical analysis was performed using SPSS software Version 21.0. (IBM Corp. Released 2012. Armonk, NY: IBM Corp) and Graph Pad Prism version 7.00 (Graph Pad Software, La Jolla, California, USA). The results were presented as mean ± standard error of mean (SEM). One-way ANOVA and Tukey tests were used for comparison of mean of measured parameters among control and treatment groups. Kruskal-Wallis, a non-parametric test, was also used to compare the reduction in percentage of parasitemia rates among different groups. Values were considered statistically significant at p<0.05.

## Results

**Figure 1:**
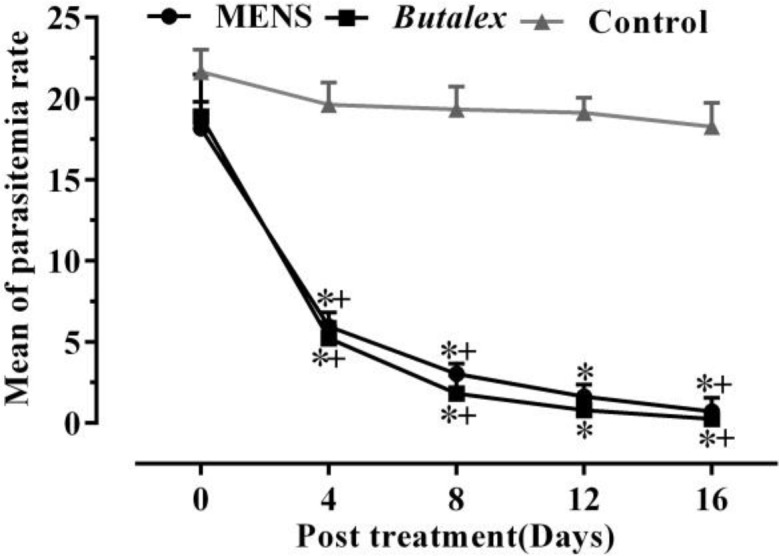
Changesinparasitemia rates following administration of *N. sativa* methanolic extract (MENS) and *Butalex*^®^ compared to control. Data are presented as mean±SEM (n=30). *Significant differences (p<0.05) between the control group versus MENS and *Butalex*^®^ groups. ^ +^Significant differences (p<0.05) between various days of each group. No significant difference was observed between MENS and *Butalex* groups.

**Figure 2:**
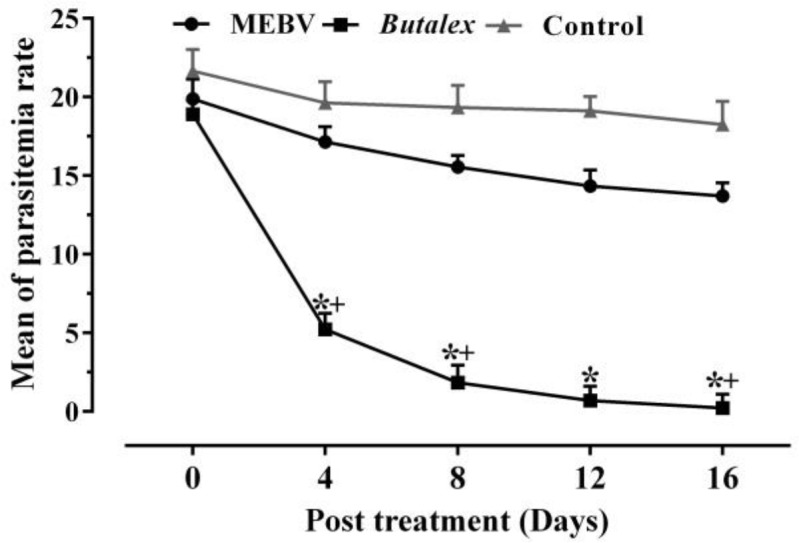
Changes in parasitemia rates following administration of *B.vulgaris* methanolic extract (MEBV) and *Butalex*^®^ compared to control. Data are presented as mean±SEM (n=30).*Significant differences (p<0.05)between the *Butalex*^®^ group versuscontrol and MEBV groups. ^+^Significant differences (p<0.05)between various days of each group. No significant difference was observed between groups MEBV and Control.

To evaluate the therapeutic effects of MENS and MEBV, a field efficacy was carried out on 120 *H. Columbae-*infected pigeons*.* Dramatically, a progressive decrease in average parasitemia rate was observed in infected pigeons treated with MENS, from18.17% before treatment to 5.96% by day 4 and 0.73% at the end of treatment (p<0.05) (Figures 1), while MEBV was unable to show significant therapeutic effects, with a maximum decrease in average parasitemia from 19.89% on the first day to 13.70% on day 16 (Figure 2) (p>0.05).

**Figure 3:**
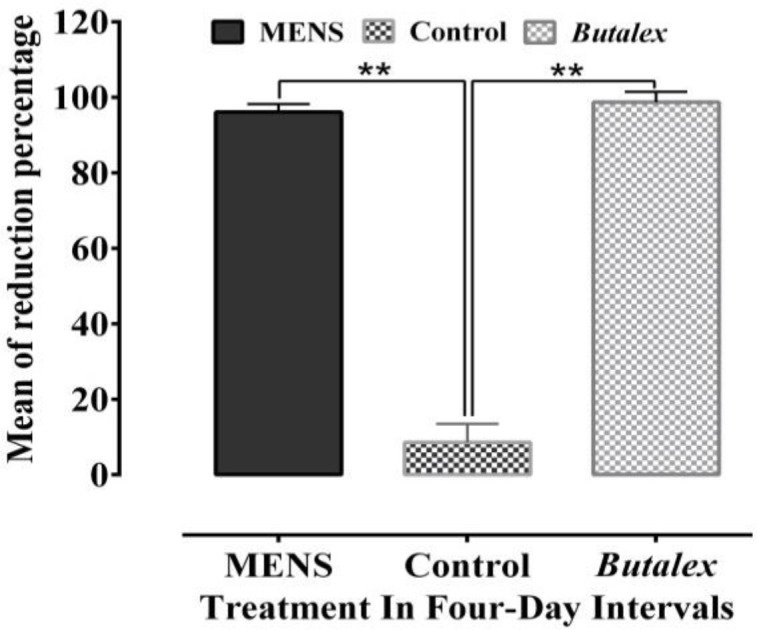
Mean of reduction percentage of parasitemia rates following treatment with *N. sativa *methanolic extract (MENS) and *Butalex*^®^. Significant differences have been shown at **p<0.001.Values are reported as Mean±SEM.

**Figure 4:**
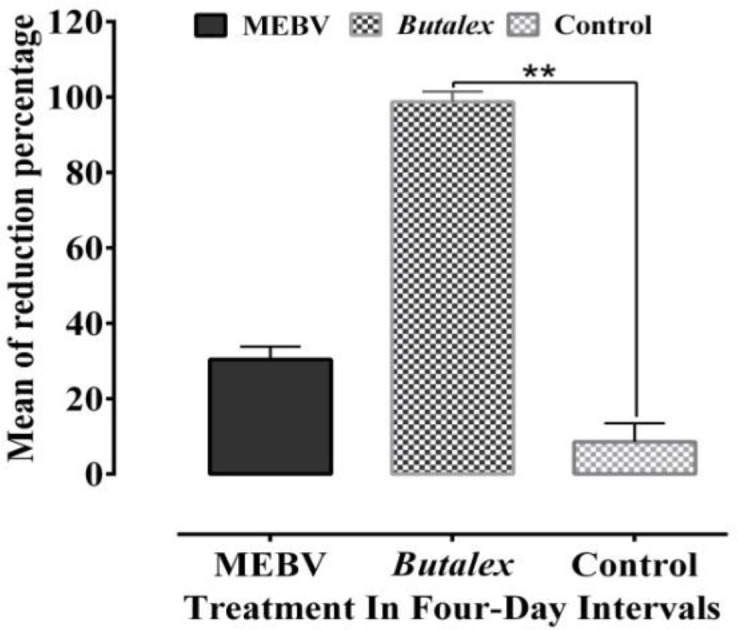
Mean of reduction percentage of parasitemia rates following treatment with *B.vulgaris* methanolic extract (MEBV) and *Butalex*^®^. Significant differences have been shown at **p<0.001.Values are reported as Mean±SEM.

The results also showed that *Butalex*^®^was able to suppress average parasitemia rate from 18.90% before treatment to 0.23% at the end of the experiment (day 16) (Figures1 and 2) (p<0.05). Figures 3 and 4 show reduction in percentage of parasitemia following treatment in all groups. Treatment of pigeons with *Butalex*^®^showed 96.19% and 98.74% reduction from day 12 to the end of experiment (day 16) (p<0.001). Compared to *Butalex*^®^*, *which is frequently used as a commercially available product, MENS-treated group with 68.02%, 84.49% and 96.14% reduction in percentage of parasitemia on days 4, 8 and 16, respectively (p<0.001) (Figures 1 and 3), showed highly effective therapeutic effectsin a time-dependent manner. Between group analysis showed a significant difference in reduction percentage of parasitemia in MENS and *Butalex*^®^-treated groups compared to MEBV group (p<0.001). However, compared to control, no significant changes in parasitemia rate were evident in groups treated with MEBV (p>0.05) (Figures2 and 4).

## Discussion

The phylum Apicomplexa forms a large and cosmopolitan assemblage of protozoan parasites. Genus *Haemoproteus *includes some species that parasitize a wide variety of birds including domestic and wild ones (Knowles et al., 2010a ▶). The application of Plant-Derived Products (PDPs) such as methanolic extracts and essential oils offers highly appropriate alternative agents compared to chemical products (El Zalabani et al., 2012 ▶). These substances are commonly used not only for keeping the social life of animals, but also for issues concerning food safety and economy, because PDPs are generally inexpensive and environmentally safe for both men and animals (Razavi et al., 2015 ▶). Some attempts have been made to find more effective anti-haemosporidial compounds and some herbal products were described as effective anti-malarial remedies (Okeola et al., 2011 ▶)*. *In the present study, the possible therapeutic activities of MENS and MEBV against *H. columbae *in naturally-infected pigeons were investigated*. *Our results showed that treatment with MENS could significantly reduce the percentage of parasitemia rates by 96.14% (p<0.001) compared to 98.74% obtained by *Butalex*^®^at the end of treatments. Our data revealed a time-dependent pattern for the therapeutic effect of MENS. At the beginning of the study, mean of parasitemia rate in MENS-treated group was 18.17±4.33 which decreased to 0.73±1.01 on day 16 post treatment (p<0.05).This therapeutic activity of MENS was in accordance with that reported against *P. yoelii* infection (Okeola et al., 2011). Although *B. vulgaris *showed anti-leishmainal and anti-malarial activity in murine models (Fata et al., 2006 ▶; Salehabadi et al., 2014 ▶), in our study, MEBV showed a weak therapeutic activity against *H. columbae* which may be due to differences in final and intermediate host, transmission, and biology of these two parasites (Zhang et al., 2014 ▶). It is possible that higher doses of MEBV or even treatment of pigeons for a period longer than 16 days, may show better therapeutic effects. *N. sativa* has been studied as a natural medicine for its biological activity and therapeutic potential against various diseases (Ahmad et al., 2013 ▶)*.* Based on the previous investigations, MENS has a high antioxidant activity and can protect rat hepatocytes against oxidative damages (Okeola et al., 201 ▶1). Nowadays, many synthetic drugs such as *Butalex*^® ^are used to treat hemoparasites but they are expensive and possess narrow margins of safety and there is a risk of drug resistance (Cheesman, 2000 ▶).To reduce the side effects of conventional drugs, some works have been conducted to use natural drugs with minimal side effects (Ahmad et al., 2013; Rahman et al., 1999)*. *Some previous studies which used MENS against different parasitic agents such as *P. yoelii *(Okeola et al., 2011a ▶), *Schistosoma mansoni *(Mahmoud et al., 2002 ▶) and *Cryptosporidium parvum *(Nasir et al., 2013 ▶) suggested that *N. sativa* has a broad-spectrum anti-parasitic activity and can be used as an alternative treatment with a different mode of action, and minimal sideeffects*. *Some investigations confirmed that applying *N. sativa* seed extract via either oral or intraperitoneal route, produces a low level of cytotoxicity in rat and mouse models (Ahmad et al., 2013 ▶; Zaoui et al., 2002 ▶)*.*Therefore, it could be concluded that MENS is safer for mammalian cells, considering that even at high concentrations no significant cytotoxicity was observed in the host cells (Mahmoudvand et al., 2014 ▶; Salem and Hossain, 2000 ▶). Although the exact mechanism via which MENS affects the infectious agents is not completely understood, some studies were performed to elucidate its mechanisms of actions. Recent investigations suggested that antimicrobial effects of MENS are attributed to itsmost important bioactive ingredients, particularly thymoquinone and other important components such as thymohydroquinone (TQ), dithymoquinone, carvacrol and p-cymene (Ahmad et al., 2013 ▶; Mahmoudvand et al., 2014 ▶; Okeola et al., 2011 ▶). TQ has been shown to suppress the Fe-NTA- induced oxidative stress and many pathological changes in Wistar rat models (Khan and Sultana, 2005 ▶). 

Majdalawieh et al. (2010) ▶ confirmed the potential immunomodulatory and anti-inflammatory effects of *N. sativa* in BALB/c mice. They reported that the aqueous extract of *N. sativa* induced Th_2 _versus Th_1 _cytokines secretion by splenocytes, while stimulation of pro-inflammatory mediators was suppressed significantly.

In conclusion, the present study confirmed that *N. sativa* has an effective anti-haematozoalproperty with unknown mode of action. Therefore, *N. sativa* may be a good candidate for developing new anti-protozoal drugs. Further studies are required to elucidate the exact mechanism(s), the mode(s) of action, and probable side effects of MENS, and investigate the application of its effective constituents. 
